# Interprofessional Experiences and Roles of Healthcare Professionals in Early Support for Semantic Variant Primary Progressive Aphasia: A Qualitative Study

**DOI:** 10.1111/1460-6984.70355

**Published:** 2026-10-16

**Authors:** Tamami Shiba, Miyae Yamakawa, Yoshimi Endo, Kenjiro Komori, Kaori Umezaki, Yasushi Takeya, Kazue Shigenobu, Satoshi Tanimukai

**Affiliations:** ^1^ Graduate School of Medicine Division of Nursing Ehime University Toon City Ehime Japan; ^2^ Graduate School of Medicine Division of Health Sciences Osaka University Suita City Osaka Japan; ^3^ Faculty of Nursing Graduate School of Nursing Science Tottori College of Nursing Kurayoshi City Tottori Japan; ^4^ Jyuzen Yurinoki Hospital Niihama City Ehime Japan; ^5^ Department of Nursing Osaka Aoyama University Minoh City Osaka Japan; ^6^ Asakayama General Hospital Sakai City Osaka Japan

**Keywords:** allied health professionals, communication support, interprofessional care, nursing, semantic dementia, svPPA

## Abstract

**Background:**

How nurses, occupational therapists, psychologists, and speech‐language therapists in Japan understand their respective roles and collaborate, particularly in the early stages of semantic variant primary progressive aphasia, has not been sufficiently investigated.

**Aims:**

To clarify the experiences and professional roles of healthcare providers supporting individuals with semantic variant primary progressive aphasia and their families during the disease's early stages.

**Methods & Procedures:**

A qualitative descriptive study using a content analysis approach was conducted. Semi‐structured interviews were conducted with ten healthcare professionals who had experience providing care to individuals with semantic variant primary progressive aphasia and their families. Interviews were audio‐recorded, transcribed verbatim, and analyzed inductively using qualitative content analysis. Data were collected in hospital‐based outpatient settings in Japan between October 2018 and April 2020.

**Outcomes & Results:**

Four main categories were identified: (1) empathic understanding of the challenges individuals with semantic variant primary progressive aphasia and their families face in comprehending the disease and the need for structured rehabilitation; (2) supporting daily routines and family communication through structured rehabilitation; (3) promoting emotional stability and continuity of daily living through early intervention; and (4) connecting professional expertise across disciplines to support individuals based on a shared understanding. Participants described the challenges families face in accepting the need for early intervention and emphasized the importance of multidisciplinary collaboration in addressing emotional, communicative, and functional difficulties.

**Conclusions & Implications:**

Care for individuals with semantic variant primary progressive aphasia requires early, structured rehabilitation embedded within a multidisciplinary framework. Clear role delineation and shared understanding among healthcare professionals are essential for effective support. Strengthening interprofessional collaboration and establishing accessible pathways for early support may help maintain daily functioning, reduce caregiver burden, and improve quality of life.

**WHAT THIS PAPER ADDS:**

*What is already known on this subject*

- Semantic variant primary progressive aphasia (svPPA) is a progressive neurodegenerative condition characterized by semantic impairments that significantly affect communication and daily functioning. Previous studies have highlighted the importance of early diagnosis and multidisciplinary involvement in supporting people with svPPA and their families. Research has also described the roles of individual professions in dementia care. Internationally, multidisciplinary team (MDT) involvement is advocated to address the diverse needs of people with young‐onset dementia, emphasizing age‐appropriate services and continuity of care. However, limited qualitative research has examined how healthcare professionals from different disciplines collaborate in the early stages of svPPA and how their roles are experienced in practice.

*What this study adds to the existing knowledge*

- This study provides an in‐depth qualitative exploration of interprofessional experiences and roles in the early support of people with svPPA. It highlights how healthcare professionals from different disciplines perceive their responsibilities, collaborate across professional boundaries, and adapt their practices to meet the complex needs of individuals with svPPA and their families. The findings reveal both facilitators and challenges of interprofessional collaboration in early svPPA care, offering new insights into role coordination, communication, and shared decision‐making that have been underexplored in previous research.

*What are the clinical implications of this study?*

- The findings underscore the importance of early and coordinated interprofessional collaboration in svPPA care. Clarifying professional roles and fostering effective communication among healthcare professionals may enhance continuity of care and support for individuals with svPPA and their families. The study suggests that nurses and allied health professionals play key roles in bridging disciplines and responding to evolving needs. These insights can inform the development of interprofessional education, service models, and clinical guidelines aimed at improving early support for people living with svPPA.

## 1 Introduction

Frontotemporal dementia (FTD) is a neurodegenerative disorder characterized by the degeneration of the frontal and temporal lobes. It includes behavioral variant frontotemporal dementia (bvFTD), which primarily presents with behavioral and personality changes, and primary progressive aphasia (PPA), which is characterized mainly by language impairment. PPA is a neurodegenerative disorder characterized by a gradual decline in language ability and is clinically classified as a language variant of FTD. It was first reported by Mesulam in 1982 and later classified into three variants by Gorno‐Tempini et al. in Gorno‐Tempini et al. 2011. One of these variants, the semantic variant (svPPA), corresponds to what was previously known as semantic dementia (SD). This study uses the term svPPA consistently. Although studies on prevalence are limited, the combined prevalence of clinical syndromes associated with frontotemporal lobar degeneration (FTLD)—including FTD, progressive supranuclear palsy, and corticobasal syndrome—is estimated at 10.8 per 100,000 people (Coyle‐Gilchrist et al. 2016). svPPA is recognized as one of the major clinical variants within this FTLD spectrum.

Caring for individuals with young‐onset dementia such as FTD is complex. Unlike typical Alzheimer's disease, FTD presents distinct symptoms, including behavioral changes and financial or social challenges that particularly affect younger families, making a one‐size‐fits‐all care model inadequate (Loi et al. 2023; Stamou et al. 2022). Internationally, multidisciplinary team (MDT) involvement is advocated to address the diverse needs of people with young‐onset dementia, emphasizing age‐appropriate services and continuity of care (Loi et al. 2023; Stamou et al. 2022). In the early stages of svPPA, when individuals present with chief complaints, such as difficulty finding words or difficulty understanding word meanings, structured rehabilitation strategies that capitalize on preserved abilities may help maintain daily communication and routines (Rogalski et al. 2025; Wauters et al. 2024). Recent systematic reviews and clinical trials support the efficacy of early, tailored interventions in preserving functional communication and enhancing participation for individuals with PPA (Rogalski et al. 2025; Wauters et al. 2024; Pagnoni et al. 2021). Such interventions can help compensate for progressive language impairments and mitigate socially challenging behaviors (Tanabe et al. 1999; Lough and Hodges 2002).

Speech‐language therapists are instrumental in rehabilitation in the absence of effective pharmacological treatment. Previous studies have demonstrated that speech‐language interventions may improve communication outcomes and communication participation in people with PPA, although the degree of efficacy varies according to intervention type, intensity, dosage, and outcome measures (Pagnoni et al. 2021; Rogalski et al. 2025). In some cases, intervention influences generalization and long‐term effects (Pagnoni et al. 2021). However, svPPA is a progressive disease. Systematic reviews indicate that speech‐language interventions for PPA predominantly include impairment‐based naming interventions, which may incorporate restorative, compensatory, or both approaches (CADÓRIO et al. 2017; Pagnoni et al. 2021; Wauters et al. 2024). According to recent randomized controlled trial evidence, communication skills training, communication partner interventions, and participation‐focused approaches are needed to enhance everyday communication and functional participation (Rogalski et al. 2025; Volkmer et al. 2020). Clinical consensus studies (Kindell et al. 2015) indicate that supporting communication in daily life and maintaining social relationships are priorities in svPPA care. In this context, dementia care requires an interdisciplinary team approach that prioritizes individuals and their families. Research on healthcare professionals’ experiences of supporting individuals with svPPA and their families is scant. Although qualitative research on communication in dementia care has focused predominantly on family perspectives, these studies have repeatedly highlighted gaps in the evidence base for communication support, particularly regarding how such support is provided and experienced across professional roles (Alsawy et al. 2017; Braithwaite et al. 2022). Despite growing recognition of the importance of communication in maintaining relationships and quality of life, the experiences of healthcare professionals involved in providing communication support remain underexplored. Although Kindell et al. (2015) provided important insights into the specialist practices of speech and language therapists who support individuals with SD, existing research has largely been limited to specific professional groups. Studies involving multiple healthcare professions have tended to focus on communication training or intervention outcomes rather than on professionals’ experiential knowledge and role perceptions in everyday clinical practice (O'brien et al. 2018). Consequently, the understanding of how nurses and other allied healthcare professionals experience and enact their roles in communication support for people with dementia and their families remains limited. Recent reviews continue to identify persistent gaps in the literature on communication‐focused care across professional roles in dementia care settings (Dhanda and Scaria 2025).

One notable exception is the work of Kindell et al. (2015), who explored everyday clinical practice among specialist speech and language therapists supporting individuals with SD and their families. While this study provides valuable insights into professional priorities and practice‐based expertise, research examining interprofessional experiences in svPPA across nursing and allied health disciplines remains scarce. Consequently, little is known about how different professionals perceive their roles, collaborate across disciplines, and navigate the challenges of supporting individuals with svPPA and their families in routine clinical practice, particularly during the early stages of the disease. Moreover, although nurses frequently interact with individuals with svPPA and their families, research examining their specific roles and how these roles are perceived by other healthcare professionals remains limited. Although the importance of collaborative, individualized care for individuals with svPPA is widely recognized, the specific contributions of each professional and the processes underpinning interprofessional collaboration remain poorly understood.

Dementia care extends beyond traditional medical management and requires multidisciplinary collaboration to address cognitive, functional, psychological, and caregiver needs (Grand et al. 2011). Recent studies indicate that coordinated multidisciplinary support and ongoing post‐diagnostic services are essential for people with young‐onset dementia to maintain their quality of life (Stamou et al. 2022). In Japan, national dementia policy promotes person‐centered care, early diagnosis and support, and coordinated multidisciplinary collaboration across medical, long‐term care, and community services (Ministry of Health, Labour, and Welfare 2024). In addition, the Clinical Practice Guideline for Dementia 2017 recommends multidisciplinary assessment, coordinated care, and continuous support tailored to the needs of people living with dementia and their families (Japanese Society of Neurology 2017). Within this multidisciplinary framework, healthcare professionals, including speech‐language therapists, psychologists, occupational therapists, and nurses, contribute complementary expertise in communication support, psychosocial care, behavioral management, family support, and ADLs. Previous research conducted in Japan has also shown that individuals with svPPA and their families often experience difficulties accessing disease‐specific information and specialized services during the early stages of the disease, highlighting the importance of coordinated multidisciplinary support and clear explanations of the purpose of rehabilitation (Shiba et al. 2023). Nevertheless, little is known about how these healthcare professionals understand their respective roles and collaborate, particularly in the early stages of svPPA. Therefore, this study aims to clarify the experiences and roles of speech‐language therapists, psychologists, occupational therapists, and outpatient nurses in supporting individuals with svPPA and their families.

## 2 Methods

### 2.1 Design

This study adopts a descriptive qualitative research design and follows the Standards for Reporting Qualitative Research (SRQR) checklist (O'brien et al. 2014) (see Supplementary Information).

### 2.2 Recruitment

Participants were recruited through convenience sampling using a regional multidisciplinary professional network involving the research team, comprising healthcare professionals involved in the care of individuals with PPA and FTD. Potential participants were informed about the study through email and direct verbal contact within the network. Healthcare professionals were eligible if they had clinical experience providing rehabilitation services or outpatient psychiatric care for individuals with svPPA and their families. No formal exclusion criteria were established, as all healthcare professionals who met the eligibility criteria and agreed to participate were included in the study.

### 2.3 Participants

A total of ten healthcare professionals participated in this study, including four allied health professionals (one speech‐language therapist, two psychologists, and one occupational therapist) and six nurses. Participant characteristics are presented in Table 1. Because svPPA is a rare condition, relatively few speech‐language therapists and occupational therapists in Japan have direct clinical experience providing rehabilitation for this population. Some nurses worked in the same hospital, whereas others provided outpatient or community‐based services, thereby contributing perspectives from diverse care settings. In the Japanese healthcare system, the care cascade for individuals with svPPA typically begins with consultations in psychiatric services, followed by neuroimaging and neuropsychological assessments conducted by clinical psychologists. Patients are then referred to outpatient follow‐up or rehabilitation delivered through home‐ or community‐based services covered by the long‐term care insurance system. Speech‐language therapists assess impairments in language and semantic memory and provide communication‐focused rehabilitation. However, therapists with specialized expertise in svPPA are limited, and access to rehabilitation services varies considerably across regions. Consequently, rehabilitation may also be provided by occupational therapists or care staff working in day care or home visit services, as well as by psychologists during outpatient visits. These interventions aim to preserve language abilities, which are considered critical for maintaining activities of daily living (ADLs). Speech‐language therapists, occupational therapists, and psychologists conduct regular functional and neuropsychological assessments and provide behavioral, cognitive, and psychosocial support. Outpatient nurses play a central role in symptom monitoring, care coordination, and patient and family education and support. Together, these professionals contribute to early intervention and the continuity of care for individuals with svPPA.

**TABLE 1:** Participant descriptions.

Participants		Professionals	Sex	Age, Mean (min–max)	Work experience, Mean (min–max)
Therapist	1	Clinical psychologist	M	48 (37–60)	23 (16–36)
	2	Clinical psychologist	M		
	3	Speech‐language therapist	F		
	4	Occupational therapist	F		
Nurse	5	Registered nurse	F	54 (40–60)	33 (20–39)
	6	Psychiatric mental health nursing specialist	F		
	7	Certified dementia nursing specialist	M		
	8	Certified dementia nursing specialist	F		
	9	Certified dementia nursing specialist	F		
	10	Certified dementia nursing specialist	F		

### 2.4 Data Collection

After receiving information about the study, participants provided written informed consent. A psychiatric nursing researcher conducted semi‐structured interviews using an interview guide (Table 2). The interview focused on the significance of supporting individuals with svPPA and their families, their perceived needs and challenges, collaboration with professionals from other disciplines, and the participants’ own roles as professionals. Representative questions included, *What do you think language rehabilitation and outpatient visits mean to individuals with early‐stage svPPA and their families?* and *What is your own role in interprofessional collaboration?*

**TABLE 2:** Interview guide.

Interview guide
In what situations and settings do you engage with individuals with early‐stage svPPA and their families? What do you think language rehabilitation and outpatient visits mean to individuals with early‐stage svPPA and their families? How do you perceive the understanding and reactions of individuals with early‐stage svPPA and their families toward language rehabilitation or outpatient visits? What kinds of difficulties or dilemmas do you experience in supporting individuals with early‐stage svPPA and their families? What strategies or efforts do you make to explain the condition, promote understanding, and provide information to individuals with early‐stage svPPA and their families? What is your own role in interprofessional collaboration, and what do therapists expect from nursing professionals in this context?

svPPA: semantic variant primary progressive aphasia.

Data were collected in hospital‐based and community outpatient settings in Japan, between October 2018 and April 2020. Interviews were conducted either in a private room at the participants’ workplace or via an online videoconferencing platform, depending on participant preference. Two interviews were conducted online during the COVID‐19 pandemic. All interviews were digitally audio‐recorded with participants’ permission. Field notes were taken during each interview to document contextual information and nonverbal observations. Each participant completed a single interview lasting approximately 40 min.

### 2.5 Researcher Background

The interviews were conducted by a psychiatric nursing researcher with clinical and qualitative research experience. Although the researcher was not employed at the institutions where the participants worked, the researcher had previous professional relationships with some participants. To ensure consistency, all interviews were conducted by the same researcher.

### 2.6 Data Confidentiality and Security

All interview data were managed in accordance with institutional confidentiality policies. Audio recordings and transcripts were stored on password‐protected, encrypted drives accessible only to the research team. Personal identifiers were removed during transcription, and pseudonyms were used to protect the participants’ privacy. If a participant requested that an interview be paused or discontinued, the researcher offered to reschedule the session to ensure voluntary participation.

### 2.7 Participant Identification

Each participant was assigned a unique identification code (e.g., Participants 1–10), which was used when presenting quotations to ensure traceability while protecting confidentiality.

### 2.8 Data Analysis

Data were analyzed inductively using qualitative content analysis (Elo and Kyngäs 2008) with NVivo 12Pro (QSR International, Doncaster, Victoria, Australia). Qualitative content analysis was selected because the aim of this study was to describe and organize healthcare professionals’ experiences into categories representing their perceptions and roles rather than to identify interpretive themes across the dataset. Unlike thematic analysis, qualitative content analysis systematically organizes data into categories that remain closely connected to the original data while allowing for progressive abstraction. Verbatim transcripts were read repeatedly and independently by one of the researchers (ST) before initial coding. Codes were generated inductively from descriptive segments of the transcripts. In accordance with Elo and Kyngäs (2008), codes with similar meanings were grouped into subcategories. These subcategories were subsequently abstracted into generic categories representing broader conceptual patterns across participants’ experiences. Finally, related generic categories were integrated into overarching main categories that represented the principal findings of the study. To enhance analytical rigor and consistency, the coding and categorization process was reviewed and refined through iterative discussions among four researchers (ST, MY, EY, and KU). To improve transparency, Table S1 presents an example of the analytical process, illustrating how an interview quotation was transformed into an initial code and subsequently abstracted into a subcategory, a generic category, and finally a main category.

### 2.9 Trustworthiness

To enhance the trustworthiness of the findings, the interview transcripts were analyzed under the supervision of researchers with expertise in psychiatric and gerontological nursing. The coding and categorization processes were discussed repeatedly among the research team to enhance analytical rigor and consistency. In addition, the preliminary categories were reviewed with gerontological nursing researchers and dementia specialists who had experience supporting individuals with svPPA and their families. These discussions served as peer debriefing and contributed to refining the interpretation of the findings. These strategies were implemented in accordance with recommendations for ensuring trustworthiness in qualitative research.

### 2.10 Ethical Considerations

This study was conducted in accordance with the Declaration of Helsinki and was approved by the Research Ethics Committees of the appropriate institutions. Written informed consent was obtained from all participants before data collection. Participation was voluntary, and participants could withdraw at any time without consequence. This qualitative descriptive study was conducted and reported in accordance with the SRQR. The SRQR checklist was used to guide data collection, analysis, and reporting (see Supplementary Information).

## 3 Results

### 3.1 Categories

Analysis of the interview data generated four main categories describing healthcare professionals’ experiences supporting individuals with svPPA and their families. These comprised 10 generic categories and 28 subcategories. The analytical process involved grouping codes into subcategories, abstracting these into generic categories, and finally integrating them into main categories. Table 3 presents the hierarchical structure of the categories.

**TABLE 3:** Hierarchical structure of the findings.

Main categories	Generic category	Subcategories
Empathic understanding of the challenges faced by individuals with svPPA and their families in understanding the disease and the need for structured rehabilitation	1–1. Difficulty understanding the need for structured rehabilitation	Hesitation to initiate rehabilitation
	1–2. Recognizing the challenges experienced by individuals with svPPA and their families	(1) The challenges perceived by healthcare professionals do not necessarily coincide with those experienced by individuals with svPPA and their families. (2) It is difficult for families to understand the disease. (3) Providing detailed explanations may sometimes increase anxiety about the future. (4) Because svPPA differs from the general image of dementia, individuals and their families may not recognize it as a disease in the early stages
2. Supporting daily routines and family communication through structured rehabilitation	2–1. Supporting daily routines through speech‐language rehabilitation	(1) Establishing daily routines through structured rehabilitation (2) Although language training has limitations, its content can be modified or replaced (3) Establishing sustainable daily routines by routinizing the practice of structured rehabilitation and regular outpatient visits
	2–2. Adapting to the preserved abilities of individuals with svPPA and their family contexts	(1) Understanding the characteristics of svPPA, the individual's personality, and preserved abilities to build a therapeutic relationship. (2) Communicating approaches to interacting with the individual according to the stage of disease progression and the family's level of understanding (3) Exploring the words or messages that the family wishes to preserve in preparation for disease progression (4) Encouraging the family to remain supportive
	2‐3. Structured rehabilitation that contributes to maintaining communication between individuals with svPPA and their families	(1) Identifying desirable behaviors that the individual is still capable of as the most important form of support (2) Recognizing the necessity of structured rehabilitation as something that should be understood by the individual, family, and healthcare professionals alike
3. Promoting emotional stability and continuity of daily living through early intervention	3–1. Importance of early intervention	(1) Intervention is most effective when initiated during the early stages of the disease (2) Early intervention is aimed at supporting long‐term home living
	3–2. Structured rehabilitation and outpatient visits as sources of emotional and life stability for individuals with svPPA and their families	(1) Structured rehabilitation contributes to emotional stability for individuals with svPPA and their families (2) Structured rehabilitation enhances understanding of symptoms and helps maintain motivation for daily life for individuals with svPPA and their families (3) Outpatient visits serve as a source of emotional support for individuals with svPPA and their families, offering access to information and someone to consult
	3–3. Consideration for the unseen struggles of individuals with svPPA and their families	(1) Individuals with svPPA and their families experience anxiety and conflict about the future (2) The discrepancy between the perceptions of individuals with svPPA and those around them is considered (3) Outpatient visits involve proactive engagement based on available information and anticipated future needs (4) Empathy and expressions of support are shown by understanding the disease characteristics and challenges of caregiving
4. Connecting professional expertise across disciplines to support individuals based on a shared understanding	4‐1. Interprofessional support based on professional expertise	(1) Communication strategies that do not rely on language when interacting with individuals with svPPA (2) Detecting changes in the physical or emotional condition of individuals with svPPA and their families, and providing appropriate support (3) Acting as a bridge between individuals with svPPA, their families, and other related professionals
	4–2. Limited information on svPPA and few specialized institutions	(1) Limited information on svPPA and few specialized institutions (2) Limited availability of therapists experienced in supporting individuals with svPPA

Abbreviation: svPPA: semantic variant primary progressive aphasia.

***Main Category 1: Empathic Understanding of the Challenges Individuals With svPPA and Their Families Face in Understanding the Disease and the Need for Structured Rehabilitation***

This category comprises two generic categories. It reflects participants’ recognition that individuals with svPPA and their families often find it difficult to understand the characteristics of the disease and may not recognize the need for early support. It also emphasizes that the challenges perceived by individuals and families do not necessarily align with those perceived by healthcare professionals. Therefore, healthcare professionals needed to respond with empathy and remain attentive to these difficulties in understanding.

***Generic Category 1–1: Difficulty Understanding the Need for Structured Rehabilitation***

Participants described how families often hesitated to initiate rehabilitation because they did not recognize the early language symptoms as manifestations of a progressive neurodegenerative disease.

Quote: “You rarely find a caregiver who is so understanding. Even when services are recommended, they may not apply for them, or they may not use the services even though they are available” (4)

***Generic Category 1–2: Recognizing That Understanding the Disease During the Early Stages Is Difficult for Individuals With svPPA and Their Families***

Participants emphasized that, before introducing rehabilitation, healthcare professionals should first understand the uncertainty, anxiety, and psychological burden experienced by individuals with svPPA and their families as they adjusted to the disease.

Quote: “From an outsider's perspective, you might think, ‘Aren't they having difficulties?’ — but the family themselves often say they're not. For example, even when the person shows abnormal eating behavior due to the disease, like putting several spoonfuls of sugar into their coffee, which clearly isn't normal, there's a kind of gap between how we see it and how the family perceives it.” (8)

Quote: “Well, we don't necessarily focus only on language rehabilitation. We also take a broader approach by asking questions such as, ‘What are your strengths?’ However, if individuals with SD say, ‘I don't need that yet’, there is little we can do.” (2)

***Main Category 2: Supporting Daily Routines and family Communication Through Structured Rehabilitation***

This category emphasizes interventions aimed at maintaining daily routines and facilitating family communication. Supporters described how rehabilitation strategies were tailored to individuals’ preserved abilities while helping families develop consistent patterns of interaction.

***Generic Category 2–1: Supporting Daily Routines Through Speech‐language Rehabilitation***

Participants emphasized that speech‐language rehabilitation plays an important role not only in improving language function but also in supporting broader aspects of everyday life, including maintaining daily routines, continuing meaningful activities, and sustaining social participation.

Quote: “Repetitive behaviors can actually be utilized. Since it becomes difficult to establish new habits as the disease progresses, it is important to start while it is still possible. Once certain behaviors are routinized, the person will continue to perform them, which can have lasting effects. The benefits of this are quite significant.” (2)

***Generic Category 2–2: Adapting to the Preserved Abilities of Individuals With svPPA and Their family Contexts***

Participants emphasized that rehabilitation should be tailored not only to the preserved abilities of individuals with svPPA but also to their family circumstances and support systems to facilitate its incorporation into everyday life.

Quote: “Well, when the disease progresses, and the person can no longer understand many things, they may end up expressing their frustration through anger. But if the family can avoid getting angry, caregiving would probably become easier. It helps when they can accept that there are simply things the person can no longer do.” (7)

Quote: “People with semantic dementia do not lose their memory itself. For example, if you show them a carrot and a peeler, they may begin peeling it automatically. So, in that sense, rehabilitation is valuable because it helps families and care providers understand that, when the environment is appropriately structured, the person is still capable of doing many things.” (4)

***Generic Category 2–3: Structured Rehabilitation That Contributes to Maintaining Communication Between Individuals With svPPA and Their Families***

Participants emphasized that structured rehabilitation helped individuals with svPPA and their families maintain communication by building on preserved abilities and fostering a shared understanding of the purpose of rehabilitation. They also described how a shared understanding among individuals, families, and healthcare professionals was essential for maintaining meaningful communication throughout disease progression.

Quote: “Since people with svPPA easily fall into routines when approached through behavioral and language‐based interventions, it's important to work on improving language‐related behaviors at home.” (8)

***Main Category 3: Promoting Emotional Stability and Continuity of Daily Living Through Early Intervention***

This category focuses on how early structured rehabilitation and consistent outpatient visits foster emotional stability among individuals with svPPA and their families. The participants described early engagement as essential for maintaining motivation and reducing anxiety.

***Generic Category 3–1: Importance of Early Intervention***

Participants emphasized that initiating structured rehabilitation during the early stages of svPPA was essential for maximizing its effectiveness and supporting individuals in remaining at home for as long as possible. Early intervention was also viewed as an opportunity to establish sustainable routines before progressive language impairment limited the acquisition of new habits.

Quote: “The challenge for individuals with semantic dementia or neurodegenerative language disorders is that without proactive involvement on our part, we cannot achieve the desired results in rehabilitation. That's what makes it so rewarding.” (4)

***Generic Category 3–2: Structured Rehabilitation and Outpatient Visits as Sources of Emotional and Life Stability for Individuals With svPPA and Their Families***

Participants described structured rehabilitation and regular outpatient visits as important sources of emotional reassurance, motivation, and practical support for both individuals with svPPA and their families.

Quote: “Just like with doctors’ explanations, it must be difficult for families without medical knowledge to understand. It's important for them to have someone who can clearly explain why rehabilitation is being done, and why the person has changed, especially when they feel, ‘This isn't the person I knew’.” (3)

Quote: “When we notice from the medical records that a caregiver seems to be becoming exhausted, we make a point of speaking with them during outpatient visits. We also let them know that they can call the nursing outpatient clinic whenever they need help or have concerns.” (10)

***Generic Category 3–3: Consideration for the Unseen Struggles of Individuals With svPPA and Their Families***

Participants emphasized the importance of recognizing the often‐unseen emotional struggles experienced by individuals with svPPA and their families and providing support based on empathy and anticipation of future needs. They highlighted the need for healthcare professionals to anticipate concerns that individuals and families might find difficult to express and to provide proactive emotional support.

Quote: “Typically, when someone suffers from aphasia due to a stroke or something similar, the inability to speak gradually becomes evident at the behavioral level. However, in the case of svPPA, the process is different. The person struggles without being able to say anything, and there's a point where their spirit breaks. I think it's important to understand that.” (7)

Quote: “The difficulties experienced by family caregivers are often invisible to others. They face many challenges that most people never encounter. As nurses, we listen carefully to their stories, acknowledge the hardships they have endured, and express our appreciation for their efforts. We do not simply tell them to ‘keep going’; rather, we encourage them by saying things like, ‘Because of you, your loved one is able to continue living at home’.” (10)

***Main Category 4: Connecting Professional Expertise Across Disciplines to Support Individuals Based on a Shared Understanding***

This category illustrates how professionals support individuals with svPPA and their families by valuing multidisciplinary collaboration that draws on complementary areas of expertise while fostering mutually supportive relationships. Participants described that, in addition to language‐focused rehabilitation, individuals with svPPA and their families required psychological support and assistance in maintaining family communication. Participants also highlighted the complementary roles of speech‐language therapists, psychologists, occupational therapists, and nurses in meeting these needs.

***Generic Category 4–1: Interprofessional Support Based on Professional Expertise***

Participants described interprofessional collaboration as drawing upon the complementary expertise of different healthcare professionals to provide coordinated and person‐centered support for individuals with svPPA and their families. They emphasized that sharing information about the individuals’ preserved strengths and meaningful activities among healthcare professionals and with family members facilitated coordinated, person‐centered care across different settings.

Quote: “As long as there is a shared understanding of the disease, I don't think it matters who takes on the management role. It could be a nurse or a therapist who is closest to the patient. Sometimes it's better to have someone, like a psychiatric social worker, explain the available support systems, so I connect them accordingly.” (8)

Quote: “The couple are no longer in a situation where they can have meaningful conversations in the usual sense. However, in their own way, they still understand each other. The husband loves singing and attends Italian singing lessons. Although he does not understand the meaning of the Italian lyrics, he is able to memorize them by rote. He has loved singing since he was young, and even without understanding the meaning, he is still able to learn and reproduce the rhythm and sound patterns of the language.” (1)

***Generic Category 4–2: Limited Information About svPPA and the Scarcity of Specialized Institutions***

Participants identified the limited availability of information, specialized services, and experienced professionals as major barriers to providing timely and appropriate support for individuals with svPPA and their families.

Quote: “Even if there are only a few, having central hubs where information is provided is very important, I think.” (3)

## 4 Discussion

This study explores the experiences and roles of therapists and outpatient nurses who support and care for individuals with svPPA and their families. The findings reveal how these professionals work collaboratively while contributing complementary expertise. Specifically, participants emphasized the importance of structured rehabilitation during the early stages of the disease to help establish daily routines and highlighted their role in facilitating collaboration across professional disciplines. A particularly important finding of this study is the discrepancy between the growing evidence supporting early intervention for PPA (Rogalski et al. 2025; Wauters et al. 2024) and the reluctance of many individuals with svPPA and their families to engage in rehabilitation during the early stages of the disease. Language impairment, the hallmark of svPPA, complicates daily communication and exacerbates caregiving challenges (Tippett and Hillis 2020; Shiba et al. 2023). Participants reported that patients and families often struggle to perceive the need for early intervention, particularly because communication is partially preserved in the early stages. Some caregivers could adapt by relying on gestures or contextual understanding, whereas others experienced anxiety regarding behavioral changes. These findings indicate that healthcare professionals should tailor support to the different ways in which families understand and respond to the disease.

Consistent with previous studies (Merrilees and Ketelle 2010), this study demonstrates that nurses provide communication and psychological support to individuals with svPPA and their families and are expected to fulfill these roles. Similarly, individuals with svPPA and their families expect speech‐language therapists not only to provide language rehabilitation but also offer psychological support and facilitate family communication (Loizidou et al. 2023). These findings reflect the significant challenges faced by individuals with svPPA and their families. Previous studies have shown that timely post‐diagnostic multidisciplinary support improves adjustment for both individuals with young‐onset dementia and their families by facilitating access to appropriate services and ongoing professional guidance (Loi et al. 2023; Stamou et al. 2022). Nevertheless, our findings suggest that many families remain hesitant to engage in early intervention because they have difficulty recognizing svPPA as a progressive neurodegenerative disease and understanding the purpose of rehabilitation. Accordingly, recommending early intervention alone is unlikely to be sufficient. Healthcare professionals should also help families understand the diagnosis, recognize the progressive nature of the condition, and appreciate the purpose and potential benefits of rehabilitation before they are ready to engage with early support.

Furthermore, although language impairment is the defining clinical feature of svPPA, behavioral, emotional, and personality changes may also emerge during the early stages of the disease and place additional strain on family relationships and caregiving (Hardy et al. 2024). These symptoms may require psychological support, behavioral management, and family counseling in addition to communication‐focused rehabilitation. This broader clinical picture strengthens the rationale for multidisciplinary care and is consistent with our findings that participants emphasized complementary roles across speech‐language therapists, psychologists, occupational therapists, and nurses to address the diverse and evolving needs of individuals with svPPA and their families.

Therefore, promoting information sharing and mutual understanding among healthcare professionals involved in the care of individuals with svPPA and their families is crucial for effective multidisciplinary practice. A systematic review by Jackson et al. (2016) identified only limited evidence regarding the effectiveness of interprofessional education in dementia care, although several studies suggested improvements in collaborative knowledge, skills, and attitudes among healthcare professionals. Since then, research has expanded, with more recent studies emphasizing collaborative learning and practice models that strengthen team‐based dementia care (Farrell et al. 2018; Lee et al. 2022; Kleinke et al. 2022; Aguirre et al. 2023). More recently, Gallée et al. (2024) highlighted the continuing need for professional development and interdisciplinary collaboration to improve care for individuals with PPA across different healthcare systems. Building on this evidence, our findings demonstrate the complementary contributions of speech‐language therapists, psychologists, occupational therapists, and nurses in supporting individuals with svPPA and their families in everyday clinical practice, thereby reinforcing the importance of interdisciplinary collaboration.

Several context‐specific implications for Japan's healthcare and long‐term care insurance systems emerge from these findings. Access to specialized speech‐language rehabilitation for svPPA remains uneven across regions because of the limited number of trained specialists. This highlights the need to develop regional clinical hubs and reinforce referral pathways between hospitals, outpatient psychiatric services, and community‐based providers. Furthermore, outpatient nurses, psychologists, and occupational therapists, who have frequent contact with families, should receive structured training to enhance their competencies in communication support and early intervention for svPPA. Coordinated informational support, including shared educational materials and joint counseling sessions, may also help families better understand the diagnosis and rationale for early rehabilitation. Reinforcing these system‐level collaborative practices within Japan's multidisciplinary care framework may enhance continuity of care and reduce the psychological and practical burden experienced by families.

### 4.1 Implications for Clinical Practice

Our findings have several implications for clinical practice. Clinical practice should prioritize interprofessional education and training to help healthcare professionals understand one another's expertise and complementary roles in supporting individuals with svPPA and their families. Such education should prepare healthcare professionals to provide consistent explanations about the diagnosis and the purpose of rehabilitation, facilitate timely referrals, coordinate communication and psychological support across disciplines, and provide coherent guidance to individuals with svPPA and their families. Importantly, our findings suggest that simply making early intervention available is unlikely to be sufficient. Many families experience difficulty recognizing svPPA as a progressive neurodegenerative disease and understanding the purpose of structured rehabilitation, which may lead to hesitation in engaging with rehabilitation during the early stages. Therefore, interdisciplinary teams should adopt a coordinated approach in which healthcare professionals communicate consistent, profession‐specific messages that support families in understanding the diagnosis, appreciating the value of rehabilitation, and engaging with appropriate services according to their level of understanding and readiness. Such coordinated communication across disciplines may facilitate earlier engagement with rehabilitation, strengthen families’ understanding and acceptance of the disease, and maximize the benefits of multidisciplinary support for individuals with svPPA and their families.

### 4.2 Strengths and Limitations

One strength of this study is its inclusion of multiple professional perspectives, which enhances the understanding of svPPA care compared with studies focusing on a single discipline. The qualitative design allowed for an in‐depth exploration of early‐stage support needs.

However, the study is limited by its small sample size and recruitment from a restricted geographic area, which may affect transferability. Furthermore, the experiences reported in this study may not capture the full diversity of svPPA care practices across Japan. Future research should examine family perspectives alongside those of healthcare professionals to comprehensively inform interprofessional practice.

## 5 Conclusions

This study highlights the complementary experiences of nurses and allied health professionals in supporting individuals with svPPA and their families. While families varied in their recognition of the need for early intervention, the participants consistently emphasized the importance of structured rehabilitation and interdisciplinary collaboration. The findings underscore the necessity of clarifying professional roles and strengthening interprofessional education to optimize care for individuals with svPPA. Future studies should examine these findings in larger and more diverse samples to enhance transferability. Further research is also needed to explore how interprofessional collaboration and professional roles evolve across different stages of svPPA. Overall, these findings provide practical guidance for developing coordinated multidisciplinary services that facilitate early engagement with rehabilitation, enhance communication and psychosocial support, and address the evolving needs of individuals with svPPA and their families.

## Funding

This study was supported by JSPS KAKENHI [Grant no. 22K10810].

## Ethics Statement

This study was conducted in accordance with the Declaration of Helsinki and was approved by the Research Ethics Committees of Osaka University Hospital (Approval No. 17480‐2; Date of Approval: 20 August 2018), Ehime University Hospital (Approval No. 1806013; Date of Approval: 25 June 2019), and Asakayama Hospital (Approval No. None; Date of Approval: 02 May 2018).

## Patient Consent Statement

Written informed consent was obtained from all participants before data collection. Participation was voluntary, and participants could withdraw at any time without consequence.

## Conflicts of Interest

The authors declare no conflicts of interest.

## Supporting information




**Supporting File**: jlcd70355‐sup‐0001‐SuppMat.docx

## Data Availability

The datasets generated during the current study are not publicly available due to ethical restrictions and concerns regarding participant confidentiality.
