# Supplementary material for: Interprofessional Experiences and Roles of Healthcare Professionals in Early Support for Semantic Variant Primary Progressive Aphasia: A Qualitative Study
Source: Int J Lang Commun Disord. 2026 Oct 16;61(6):e70355. doi: 10.1111/1460-6984.70355 (PMC13674717; doi:10.1111/1460-6984.70355)
Supplement: Supplementary file 1 [file JLCD-61-0-s001.docx]

**Supplementary Table 1-1**

| **Utterances** | **Coded Utterances** | **Subcategories** |
| --- | --- | --- |
| It’s really in the early stages that you can establish good habits, isn’t it? I think the early stages are key. It’s about deciding right from the start how you’re going to live your life—or rather, putting together a sort of ‘curriculum’, so to speak—and how things turn out then has a huge impact on what happens afterwards (2) | For people with svPPA and their families, the sooner intervention begins, the better | For those involved in svPPA, results can only be achieved by getting actively involved from the outset |
| The challenge for individuals with semantic dementia or neurodegenerative language disorders is that without proactive involvement on our part, we cannot achieve the desired results in rehabilitation. That’s what makes it so rewarding. (4) | svPPA does not improve on its own; the outcome will depend on the extent to which the therapist is involved in svPPA. |  |

**Supplementary Table 1-2**

| **Subcategories** | **Generic Categories** |
| --- | --- |
| For those involved in svPPA, results can only be achieved by getting actively involved from the outset | Importance of Early Intervention |
| Early intervention is aimed at supporting long-term independent living at home |  |

**Supplementary Table 1-3**

| **Generic Categories** | **Main Categories** |
| --- | --- |
| Importance of Early Intervention | Promoting emotional stability and continuity of daily living through early intervention |
| Structured rehabilitation and outpatient visits as sources of emotional and life stability for individuals with svPPA and their families |  |
| Consideration for the unseen struggles of individuals with svPPA and their families |  |
